# Supplementary material for: Impact of ceftiofur administration and Escherichia coli inoculation on the calf fecal microbiome
Source: mSystems. 2026 Jun 9;11(7):e00501-26. doi: 10.1128/msystems.00501-26 (PMC13386986; doi:10.1128/msystems.00501-26)
Supplement: Supplemental material — Supplemental legends and File S1. [file msystems.00501-26-s0006.docx]

**SUPPLEMENTAL MATERIAL**

**Figure S1:** Beta-diversity PCoA plots based on Euclidean distances of CLR-transformed MAG abundance profiles for the GWAS (**A**) and NJ (**B**) administration groups, after removing MAGs with <10% read-mapping coverage. For each administration group, two panels are shown: the top colored by treatment (green=control; orange=ceftiofur) and the bottom shaded by administration day. When all MAGs were included without the coverage filter, separation in the NJ group became less distinct, whereas patterns in the GWAS group remained largely unchanged (data not shown). **C**: PCoA of beta-diversity based on Euclidean distances of MAG abundance counts. Statistical tests and annotation are based on samples grouped by sampling timepoint (top) or animal ID number (bottom). Differences between groups were assessed using PERMANOVA, and group dispersion was evaluated using betadisper.

**Figure S2**: Identification of MAG prevalence across ceftiofur-exposed and control samples. (**A**). Heatmap showing the number of highly prevalent MAGs (>80% prevalence) unique to or shared between treatment groups. MAGs are grouped by family, or by the most specific available taxonomic level if a family-level classification was not possible. (**B**) Prevalence distribution of MAGs from each category (shared, highly prevalent in ceftiofur-exposed, highly prevalent in control) across the 25 control samples. (**C**) Prevalence distribution of MAGs from each category (shared, highly prevalent in ceftiofur-exposed, highly prevalent in control) across the 25 ceftiofur-exposed samples. (**D**) Relative abundance of MAGs (Enterobacteriaceae) across sampling days. Each point represents a sample, plotted according to its relative abundance (x-axis) and day of administration (y-axis). Points are colored by treatment (red = ceftiofur; green = control) and transparency reflects sequencing coverage (higher opacity indicates higher coverage). Jitter was applied to improve visualization of overlapping points.

**Figure S3**: Distribution of MAGs encoding ARGs with at least 90% reference gene coverage. Heatmap shows the taxonomic identification of each MAG at the family level, when available. The numbers inside the boxes indicate the number of unique MAGs in which a given gene was identified.

**Figure S4**: Normalized abundances of CAG-485 (Muribaculaceae) MAGs encoding either the *cfxA5* (top MAG) or the *cfxA6* (bottom MAG) gene across each sampling date. The *cfxA5* (AY769934) gene was encoded with 94.2% identify by a MAG classified as CAG-485; the *cfxA6* (GQ342996) gene was encoded with 91.7% identify by a separate MAG classified as CAG-485 sp017547845.

**Figure S5:** Abundance of the five ARGs present in the administered *E. coli* strains and reliably identified in the ARG abundance tables (>90% coverage) following KMA mapping. Each point represents an individual sample, plotted by sampling day and colored according to treatment group (control in green, ceftiofur in orange). Points are jittered to improve visualization of overlapping observations.

**Table S1**: Metadata for *E. coli* inoculated to calves in this study

**Table S2**: Metadata for all sequenced fecal samples in this study.

**Table S3: A:** Taxonomic summary and associated CheckM metrics of the 1607 MAGs with greater than 90% completeness and less than 5% contamination. **B***:* MAGs identified as highly enriched in either control or ceftiofur-exposed samples via *Indicspecies* analysis (IndVal>0.80; fdr adjusted p<0.05). Statistical comparisons included only samples from 05d onwards.

**Table S4:** **A:** Taxonomic identification of sequenced single-cells through classification of genome k-mer signatures against the GTDB database. **B:** Accession numbers for the SCS data.

**Supplementary File 1: Identification of E. coli-derived contigs and potential HGT events.** Mapping between genomes of administered *E. coli* genomes and denovo assembled contigs. Local BLAST Comparisons were used to identify: A) qualitatively and quantitatively the presence of the administered *E. coli* strains in our samples, B) chimeric contigs indicating potential Horizontal Gene Transfer events. Detailed descriptions of the methods and the results are provided, as well as two tables summarizing the results of A and B.

### ****Methods****

Assembled contigs longer than 1 kbp from shotgun metagenomic samples were mapped to the administered E. coli genomes using Bowtie2 (Hector consortium, accession numbers; Table 1). Succesfully mapped contigs were further used for BLAST searches against genome sequences of the administered cocktail strains available at NCBI. Contigs shorter than 2 kbp, with less than 98% identity, or containing more than one gap or mismatch per 1,000 bp were filtered out (in accordance with the Illumina platform error rate).

Subsequently, BLAST output tables were further filtered in R to identify and subset contigs containing multiple regions mapping to different E. coli genomes (by retaining only contigs that showed a partial match to the administered *E. coli* strains). Chimeric contigs were further screened using MobileElementFinder and ResFinder to identify mobile genetic elements and antimicrobial resistance genes.

### ****Results****

Fragments of administered E. coli genomes were detected in seven animals, with most contigs observed in two animals from the NJ control group on days 5 and 13 (Table 1), suggesting transient presence of some strains but without reliably identifying specific ones.

Screening for chimeric contigs revealed 38 sequences showing partial similarity to distinct genomes, which were further analyzed for antimicrobial resistance genes and mobile elements. Two contigs contained both the ***floR*** phenicol resistance gene and the **ISVsa3** insertion sequence, and one contig contained the ***sul2*** sulfonamide resistance gene with the **IS942** insertion sequence, tentatively indicating a potential horizontal transfer of resistance genes facilitated by mobile elements (Table 2).

**Table 1.** Number of contigs identified as having >98% nucleotide identity, fewer than 1/1,000 bp mismatch, and lengths >2 kbp when compared with genomes of the administered *E. coli* cocktail strains (presented on the left with their NCBI accession numbers). Column headers show the matching *E. coli* genomes by their NCBI accession numbers. Sample metadata are shown as column headers.

| **Date** | **24082020** | **30082020** | **30082020** | **7092020** | **7092020** | **7092020** | **21092020** |
| --- | --- | --- | --- | --- | --- | --- | --- |
| **Administration day** | **d-1** | **d+5** | **d+5** | **d+13** | **d+13** | **d+13** | **d+27** |
| **Group** | **ctrl** | **ctrl** | **ctrl** | **ctrl** | **ctrl** | **ceftiofur** | **ceftiofur** |
| **Animal** | **903** | **903** | **901** | **916** | **929** | **915** | **930** |
| **GCA_025621285.1** | 0 | 0 | 9 | 17 | 3 | 0 | 0 |
| **GCA_025634785.1** | 1 | 2 | 5 | 6 | 4 | 0 | 2 |
| **GCA_025636805.1** | 1 | 0 | 109 | 28 | 6 | 1 | 0 |
| **GCA_025639665.1** | 1 | 0 | 5 | 2 | 1 | 0 | 0 |
| **GCA_025640905.1** | 0 | 0 | 5 | 9 | 4 | 0 | 0 |
| **GCA_025641405.1** | 2 | 0 | 8 | 4 | 1 | 0 | 0 |
| **GCA_025641675.1** | 1 | 0 | 12 | 18 | 6 | 0 | 0 |
| **SRS9240299** | 0 | 0 | 3 | 4 | 0 | 0 | 0 |
| **SRS9240555** | 0 | 0 | 4 | 2 | 0 | 0 | 0 |
| **SRS9240557** | 0 | 0 | 3 | 5 | 1 | 0 | 0 |
| **SRS9240596** | 0 | 0 | 20 | 38 | 5 | 0 | 0 |
| **SRS9240597** | 0 | 0 | 25 | 32 | 5 | 0 | 0 |
| **SRS9240598** | 0 | 0 | 2 | 3 | 0 | 0 | 0 |
| **SRS9240642** | 1 | 0 | 9 | 13 | 3 | 0 | 0 |
| **SRS9240691** | 0 | 0 | 3 | 2 | 0 | 0 | 0 |
| **SRS9241806** | 0 | 0 | 2 | 2 | 0 | 0 | 0 |
| **SRS9241812** | 0 | 0 | 15 | 32 | 5 | 0 | 0 |
| **SRS9241886** | 0 | 0 | 2 | 11 | 1 | 0 | 0 |
| **SRS9241901** | 0 | 0 | 0 | 2 | 0 | 0 | 0 |
| **SRS9242020** | 1 | 0 | 0 | 2 | 1 | 0 | 0 |
| **SRS9242025** | 1 | 0 | 0 | 2 | 1 | 0 | 0 |
| **SRS9242026** | 0 | 0 | 0 | 1 | 0 | 0 | 0 |
| **SRS9242027** | 1 | 0 | 0 | 2 | 1 | 0 | 0 |
| **SRS9242029** | 1 | 0 | 2 | 2 | 1 | 0 | 0 |
| **SRS9242035** | 0 | 0 | 1 | 1 | 0 | 0 | 0 |

**Table 2**: Summary of the three chimeric contigs showing partial sequence identity to the *Escherichia coli* administered strains. For each contig, the identified mobile genetic element (detected using MEfinder), contig length, alignment structure (CIGAR string), and associated antimicrobial resistance (ARG) and virulence genes are reported. Resistance and virulence genes were identified by sequence similarity BLAST searches against ResFinder and VFdb.

|  | **Mobile element** | **Contig length** | **cigar** | **resistance gene** | **virulence gene** |
| --- | --- | --- | --- | --- | --- |
| **Contig 1** | IS942 | 3161 | M1140 I6 M452 | sulphomide_sul2_2 AY034138_1 | VFG013516(gb\|WP_010944247) (mrsA/glmM) phosphoglucosamine_mutase |
| **Contig 2** | ISVsa3 | 4411 | M977 | phenicol_floR_2 AF118107_1 |  |
| **Contig 3** | ISVsa3 | 4795 | M977 | phenicol_floR_2 AF118107_1 | VFG013516(gb\|WP_010944247) (mrsA/glmM) phosphoglucosamine_mutase |
